# Supplementary material for: Direct observation of a wakefield generated with structured light
Source: Nat Commun. 2025 Dec 8;16:10957. doi: 10.1038/s41467-025-66056-5 (PMC12686447; doi:10.1038/s41467-025-66056-5)
Supplement: Supplementary file 1 — Supplementary Information [file 41467_2025_66056_MOESM1_ESM.pdf]

Supplementary

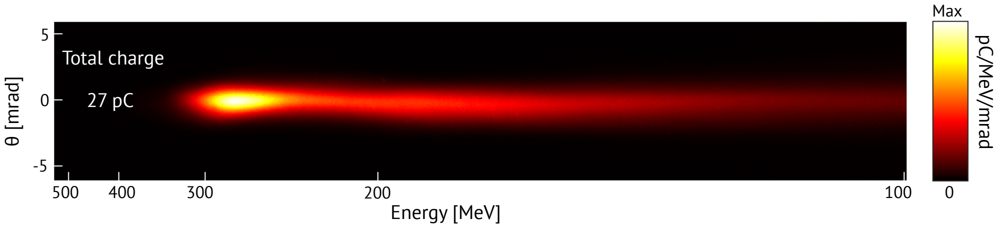

Supplementary Figure 1 Sample angularly resolved spectrum of the electrons accelerated by beam 2.

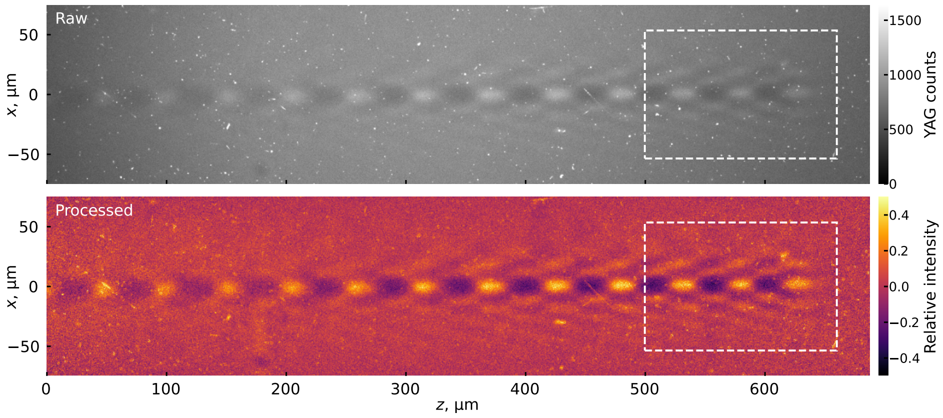

Supplementary Figure 2 Raw (top) and processed (bottom) FREM images corresponding to the case in Fig. 2(b). The white rectangle shows the crop used in Fig. 2(b).

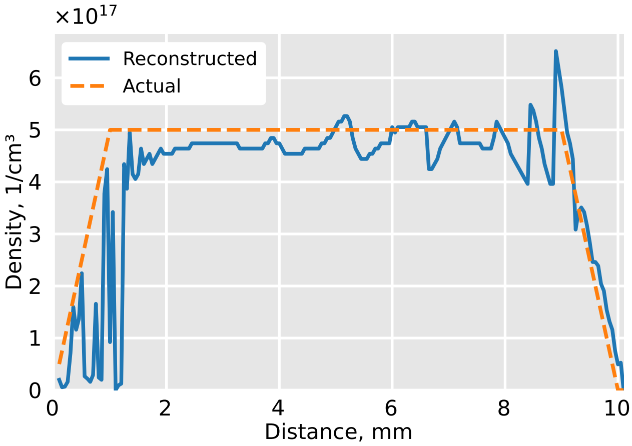

Supplementary Figure 3 Comparison of density reconstructed from simulated probe images to the density profile used in PIC simulations.

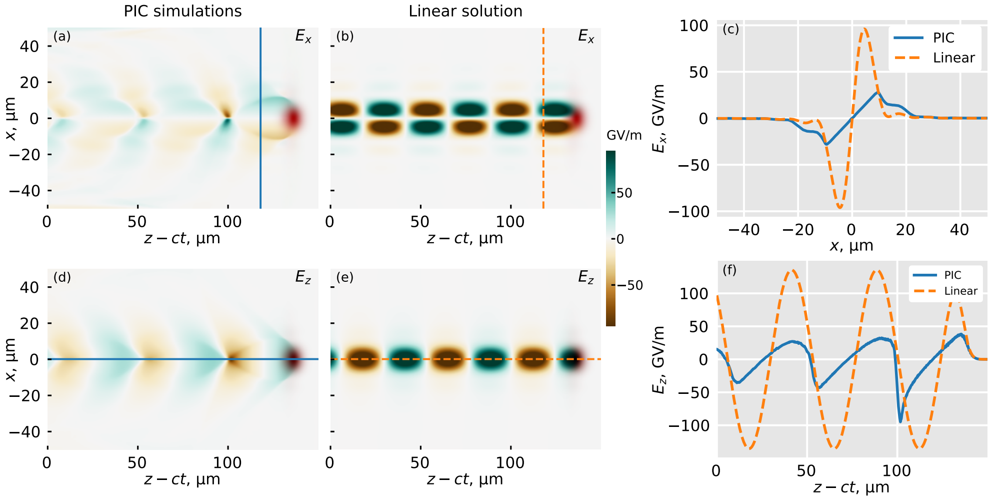

Supplementary Figure 4 Comparison of the spatial distribution of the transverse electric field E_x between (a) the PIC simulation for the parabola-reflected pulse and (b) the corresponding calculated linear solution. (c) Transverse distribution of E_x for a slice $z - ct = 118\,\mu\text{m}$ shown with vertical lines in (a–b). Comparison of the longitudinal electric field E_z for (d) the PIC simulation and (e) the linear solution. (f) On-axis longitudinal distributions of E_z. The red color in (a–b, d–e) shows the distribution of the laser pulse intensity.
